# Integrated Multiparametric Radiomics and Informatics System for Characterizing Breast Tumor Characteristics with the OncotypeDX Gene Assay

**DOI:** 10.3390/cancers12102772

**Published:** 2020-09-27

**Authors:** Michael A. Jacobs, Christopher B. Umbricht, Vishwa S. Parekh, Riham H. El Khouli, Leslie Cope, Katarzyna J. Macura, Susan Harvey, Antonio C. Wolff

**Affiliations:** 1 The Russell H. Morgan Department of Radiology and Radiological Science, The Johns Hopkins School of Medicine, Baltimore, MD 21205, USA; vishwaparekh@jhu.edu (V.S.P.); kmacura@jhmi.edu (K.J.M.); Susan.Harvey@hologic.com (S.H.); 2 Sidney Kimmel Comprehensive Cancer Center, The Johns Hopkins School of Medicine, Baltimore, MD 21205, USA; cumbrich@jhmi.edu (C.B.U.); awolff@jhmi.edu (A.C.W.); 3 Department of Computer Science, The Johns Hopkins University, Baltimore, MD 21210, USA; 4 Department of Radiology and Radiological Sciences, University of Kentucky, Lexington, KY 40536, USA; elkhoulir@uky.edu; 5 Department of Oncology, The Johns Hopkins School of Medicine, Baltimore, MD 21205, USA; lcope1@jhmi.edu; 6 Hologic Inc., 36 Apple Ridge Rd. Danbury, CT 06810, USA

**Keywords:** mpRad, radiomics, multiparametric radiomics, informatics, IRIS, machine learning, breast, magnetic resonance imaging, diffusion-weighted imaging, DWI, ADC map, cancer, OncotypeDX

## Abstract

**Simple Summary:**

Artificial Intelligence methods using machine learning and radiomics is an emerging area of research for radiological and oncological applications for patient management. Recent evidence from breast cancer suggests that different breast cancer subtypes may respond differently to adjuvant therapies. The use of a 21-gene array assay called OncotypeDX can predict potential recurrence of cancer in patients with estrogen positive breast cancer after treatment, however, there are potential cost disadvantages that hamper its widespread use. Multiparametric magnetic resonance imaging can simultaneously identify key functional parameters and provide unique imaging phenotypes of breast cancer, which is used in radiomic analysis. Radiomics provide quantitative information of different tissue types. We have developed a new machine learning radiomic informatics tool that integrates clinical and imaging variables, single, and multiparametric radiomics to compare with the OncotypeDX test to stratify patients into three risk groups: low, medium, and high risk of breast cancer recurrence.

**Abstract:**

Optimal use of multiparametric magnetic resonance imaging (mpMRI) can identify key MRI parameters and provide unique tissue signatures defining phenotypes of breast cancer. We have developed and implemented a new machine-learning informatic system, termed Informatics Radiomics Integration System (IRIS) that integrates clinical variables, derived from imaging and electronic medical health records (EHR) with multiparametric radiomics (mpRad) for identifying potential risk of local or systemic recurrence in breast cancer patients. We tested the model in patients (*n* = 80) who had Estrogen Receptor positive disease and underwent OncotypeDX gene testing, radiomic analysis, and breast mpMRI. The IRIS method was trained using the mpMRI, clinical, pathologic, and radiomic descriptors for prediction of the OncotypeDX risk score. The trained mpRad IRIS model had a 95% and specificity was 83% with an Area Under the Curve (AUC) of 0.89 for classifying low risk patients from the intermediate and high-risk groups. The lesion size was larger for the high-risk group (2.9 ± 1.7 mm) and lower for both low risk (1.9 ± 1.3 mm) and intermediate risk (1.7 ± 1.4 mm) groups. The lesion apparent diffusion coefficient (ADC) map values for high- and intermediate-risk groups were significantly (*p* < 0.05) lower than the low-risk group (1.14 vs. 1.49 × 10^−3^ mm^2^/s). These initial studies provide deeper insight into the clinical, pathological, quantitative imaging, and radiomic features, and provide the foundation to relate these features to the assessment of treatment response for improved personalized medicine.

## 1. Introduction

Integrating clinical health information with radiological imaging and other biomarkers could be beneficial for different types of cancer. This integration of seemingly disparate data may improve our understanding of the complex nature of cancer and potentially provide predictive markers with clinical benefit in certain cancer phenotypes. For example, in breast cancer, there is active research on how to predict the potential of local recurrence after conservative treatment. One such method to predict local and systemic recurrence of breast cancer is the OncotypeDX assay [1,2,3]. OncotypeDX is based on the mRNA expression by RT-PCR in estrogen receptor (ER) positive disease without the human growth factor receptor 2 (HER2-neu) overexpression and is tested on tissue obtained at biopsy or core diagnostic or surgical samples [4,5]. OncotypeDX has been validated in prospective studies as a prognostic tool predictive of excellent outcomes in patients with ER-positive disease treated with endocrine therapy [1]. It has since shown to be a predictive tool to identify patients with breast cancer, most likely to benefit from the addition of adjuvant chemotherapy to endocrine therapy [1,2,3,4,6,7,8,9,10]

Multiparametric (mp) radiological imaging can accurately detect and characterize breast lesions using advanced quantitative parameters [11,12,13,14]. Dynamic contrast-enhanced (DCE)-magnetic resonance imaging (MRI), which is a marker of the vascularity and permeability of breast lesions, can characterize malignant lesions by the rapid uptake of a contrast agent, followed by fast washout over time, and benign lesions by slow uptake and persistent or plateau washout [11,15,16,17,18,19]. Moreover, by interrogating the movement of water within the intra- and inter-cellular environments of normal and lesion tissue using diffusion-weighted imaging (DWI), with the apparent diffusion coefficient (ADC) of water map, can provide characterization of breast and other lesions [12,20,21,22,23,24]. Recent research had demonstrated that textural and shape analysis of multiparametric radiomics (mpRad) could potentially add a unique insight into the underlying tissue pathology [25]. Radiomics is an area of research, which deals with quantitative and qualitative textural analysis of the underlying tissue pathology [26,27,28,29,30,31,32,33]. Radiomic analysis of breast mpMRI has been investigated for breast cancer diagnosis and treatment response assessment in the research setting across multiple studies [25,26,27,28,31,32,34]. However, to date, no one has investigated using mpRad coupled with breast mpMRI and informatic data and testing with OncotypeDX for breast lesion characterization.

The challenge is to accurately combine mpMRI with radiomic, clinical, and pathologic features to stratify patients and identify the potential for cancer recurrence, similar to the OncotypeDX. To answer this challenge, we have developed a new machine-learning informatic method termed Integrated Radiomics Informatic System (IRIS), which can be applied to multiparametric MRI and radiomics, clinical, and pathologic descriptors, as well as a gene array analysis [32,35,36,37,38]. The purpose of this study is to test the IRIS algorithm by combining data from imaging, radiomics, and electronic medical health records (EHR) to stratify patients into three risk groups: low, medium, and high risk, and then compare these groups with the OncotypeDX 21-gene assay scores.

## 2. Methods

### 2.1. Clinical Subjects

All studies were performed in accordance with the institutional guidelines for clinical research under a protocol approved by our Institutional Review Board (IRB number: NA_00001113 and NA_00022703), and all Health Insurance Portability and Accountability Act (HIPAA) agreements were followed for this retrospective study and informed consent was waived. All procedures performed in studies involving human participants were in accordance with the ethical standards of the institutional and/or national research committee and with the 1964 Helsinki declaration and its later amendments or comparable ethical standards. Patients were selected from the Johns Hopkins Integrated Breast Cancer Research Database, developed by one on the authors (C.U.), and who had underwent MRI as part of the clinical health record review. Of the patients with biopsy proven breast cancer who presented to our facility for bilateral breast MRI, 123 patients were identified to have both the OncotypeDX and an advanced MRI exam, which included DCE and DWI. Our inclusion criteria were: (1) breast imaging on 3T MRI scanner, (2) dynamic contrast enhanced and DWI MRI sequences, and (3) pathology proven diagnosis of Estrogen receptor positive (ER+) breast cancer and (4) OncotypeDX having been performed on lesion tissue samples. There were 80 patients with 83 lesions that satisfied the inclusion criteria. 

### 2.2. Histological Phenotyping

All breast cancers were categorized by histological phenotyping based upon immunohistochemistry (IHC). Estrogen and progesterone receptors (ER and PR), HER2-neu by Fluorescence in situ hybridization (FISH), and Ki-67 proliferation index (%). The Elston tumor grades for each lesion were distributed as Grade 1 (9%), Grade 2 (78%), and Grade 3 (13%). Histopathological data was obtained from the breast pathology database (C.B.U.). All patient demographics matched the current clinical criteria for the OncotypeDX test (ER+).

### 2.3. Integrated Radiomics Informatics System (IRIS)

#### 2.3.1. Multi-Subspace Embedding and Clustering

**Figure 1:**
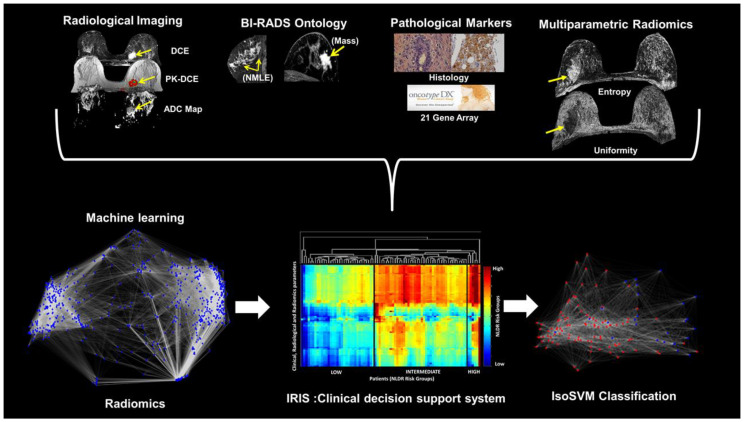
Illustration of the Integrated Radiomics Informatics System (IRIS) using different data inputs and the OncotypeDX. (**Top**) the high dimensional patient space created by the radiological characteristics, informatics, radiomics, and clinical information. (**Bottom**) IRIS transforms the patient space into (**Left**) a complex interaction network for visualization of inter-parameter relationships, (**Middle**) multi-subspace embedding, and clustering heatmap to indicate potential risk identified by each IRIS parameter and the risk signature for each patient, and (**Right**) final patient classes (e.g., low, or high-risk) classified using the combine Isomap and Support Vector Machine (IsoSVM) classification algorithm.

We have developed a machine learning model that integrates different types of clinical and imaging parameters, which allows for the construction of a clinical decision support model [36,38,39]. The inherent high dimensionality of the clinical and imaging parameters and any complex correlations within the data presents significant challenges for integration and visualization of the data. These challenges were solved using the nonlinear dimensionality reduction (NLDR) method [39]. Nonlinear dimensionality reduction algorithms transform and embed a *D* dimensional space into a lower *d* dimensional manifold representation of *D’s* intrinsic dimensionality, where *d < D*. The goal of the multi-subspace embedding and clustering method is to transform the patient space, represented as $X=\left\{x_{1},x_{2},\;\ldots ,\;x_{n_{p}}\right\}\in R^{D}$ where, *x_i_* represents the *i*th patient, *n_p_* represents the number of patients, and D represents the number of clinical and imaging parameters, into an Integrated Radiomics Informatics System (IRIS) visualized by a heatmap as shown in Figure 1. The steps of the IRIS system are outlined below [32,36]. 

In the first step, *n_p_* d-dimensional subspaces, where d∈{1,2,3}, are extracted from each D-dimensional patient vector, $x_{i}$, by selecting all combinations of one, two, or three IRIS parameters from *D*. Here, *n_p_* is given by ∁ (D, 3). Each subspace can be represented as in Equation (1):

(1) $$S_{i}=\left\{s_{1},s_{2},\ldots ,s_{n_{p}}\right\}\in R^{d}\;\forall \;d\in \left\{1,2,3\right\},\;i\in \left\{1,2,\ldots ,n_{p}\;\right\},$$

The second step involves transformation of each d-dimensional subspace, $S_{i}\in R^{d}\;\forall \;i\in \left\{1,2,\ldots ,n_{p}\right\}$ into a one dimensional embedding, $Y_{i}=\left\{y_{1},y_{2},\ldots ,y_{n_{p}}\right\}\in R^{1}\;\forall i\in \left\{1,2,\ldots ,n_{p}\right\}$ using a nonlinear dimensionality reduction algorithm [40]. In the third step, each one-dimensional embedding, *Y_i_* is evaluated against a ground truth (e.g., OncotypeDX scale) based on the correlation coefficient, *R_i_* between *Y_i_* and the ground truth. In this paper, we employed the correlation coefficient as the evaluation metric. The aim of this step is to identify the optimal set of one-dimensional embeddings, *U*, such that, *R_i_* ≥ 0.5, as shown in the following Equation (2):

(2) $$U=\{Y_{i}\in Y|R_{i}\geq 0.5\}\;\forall \;i\in \left\{1,2,\ldots ,n_{p}\right\},$$

In the fourth step, a hierarchical clustering algorithm is used to cluster the set, *U* to produce two clustering configurations. The first clustering configuration is between different informatics and radiomics parameters. The first clustering configuration provides a visualization of relationships between the clinical and imaging parameter embedding, each parameter’s importance, and identify redundant embeddings. The second clustering configuration is between each patient, visualizing the relationship between different patients. This allows IRIS to identify patients clustered into different risk groups and classify any unknown patient into a relevant risk group.

#### 2.3.2. Feature Importance

In IRIS, the importance of each clinical or imaging parameter, *i* is calculated as the percentage of embeddings in *U* that include parameter *i*. The contribution of each parameter to the IRIS model allows for assessment of which parameters to keep and which to discard.

#### 2.3.3. Complex Network Analysis of Informatics Parameters

Using IRIS, the high dimensional relationship between different clinical and imaging parameters was explored by modeling and analyzing a complex informatics network. Before modeling the complex network, the raw values corresponding to each clinical and imaging parameter were transformed into risk prediction score normalized in the range 0–1, such that zero corresponds to low risk and one corresponds to high risk. The normalized risk prediction score for every parameter was calculated using the following steps:

- First, a correlation coefficient, *r_i_* between the values, *y_i_* spanned by the parameter, *i* across all the patients and corresponding OncotypeDX scores are calculated.
- Second, the range of clinical and imaging parameter values across all the patients are normalized from zero to one according to the following formula (3):

(3) $$z_{i}=\;\{\begin{array}{c} \frac{y_{i}-\min\left(y_{i}\right)}{\max\left(y_{i}\right)-\min\left(y_{i}\right)},\;if\;r_{i}\geq 0\;\\ 1-\;\frac{y_{i}-\min\left(y_{i}\right)}{\max\left(y_{i}\right)-\min\left(y_{i}\right)},\;if\;r_{i}<0 \end{array}$$

Here, *z_i_* represents the resultant risk prediction score for each parameter, *i*.

#### 2.3.4. Construction of Network Model of Informatics Parameters

The complex informatics network, *G* was constructed from the multidimensional data, $Zi=\left\{z_{1},z_{2},\ldots ,z_{K}\right\}\in R^{n_{p}}$, where, *z_i_* represents the risk prediction score of the clinical and imaging parameter, *i*; *K* is the number of clinical and imaging parameters; and *n_p_* is the number of patients [38]. The network *G* is represented as *G* = (*V*,*E*) with *V* = {*v*_1_,*v*_2_,…,*v_K_*} being the set of *K* vertices representing the clinical and imaging parameters and *E* being the adjacency matrix indicating the interactions between the clinical and imaging parameters in the form of edge weights. The edge weight between any two vertices *v_i_* and *v_j_* was computed by Equation (4) as follows

(4) $$E_{ij}=1-corr\left(z_{i},z_{j}\right),$$

The edge weights represent the distances between any two parameters. We defined a neighborhood parameter, *k*, to define the number of nearest neighbors each parameter could be connected to. The connectivity in the resulting complex network is dependent on the value of *k* chosen. If the value of *k* is chosen to be too large, the complex network may produce short circuit or spurious connections while a low value of *k* would produce a disconnected network [40]. The value of k selected as three by empirical analysis.

#### 2.3.5. Statistics and Topological Characteristics of the Complex Network

The complex network was analyzed using graph summary metrics and centrality metrics [41,42] The average path length and diameter are the basic statistical metrics computed for any complex network. A path is defined as the set of edges connecting any two nodes and the sum of weights of these edges represent the path length. Average path length, as the name suggests, is the average of the path lengths across all pairs of nodes or clinical and imaging parameters. Diameter is the maximum value among all the path lengths. Graph centrality metrics identify the most important clinical and imaging parameters in the complex network and called the hub nodes [43,44]. These hub nodes influence the network properties. Furthermore, the probability of any incoming node connecting to these hub nodes is significantly higher than connecting to other nodes [42]. The hub nodes may correspond to the key clinical and imaging parameters that are predictors of breast cancer recurrence risk. In total, the following metrics were extracted from the complex network: degree distribution, average path length, diameter, clustering coefficient, and different centrality measures such as degree centrality, harmonic centrality, and betweenness centrality (see Appendix A). 

#### 2.3.6. Multiparametric and Single Radiomics

##### The Multiparametric Radiomic Tissue Signature Model

We define a tissue signature (TS) that represents the composite feature representation of a tissue type based each of the different imaging sequences [25]. Mathematically, for *N* different imaging parameters with TS at a voxel position, *S_p_* is defined as a vector of gray level intensity values at that voxel position, *p* across all the (*N*) images in the data sequence for different tissue types and is given by the Equation (5),

(5) $$S_{p}=\;{\left[I_{p}^{\left(1\right)},I_{p}^{\left(2\right)},I_{p}^{\left(3\right)},\;\ldots ,I_{p}^{\left(N\right)}\right]}^{T},$$

where, *I_p_* is the intensity at voxel position, p on each image, and *T* is the transpose. Then we define the tissue signature probability matrix (TSPM) as an *N* dimensional matrix with each cell representing a tissue signature configuration. The TSPM characterizes the spatial distribution of tissue signatures within a Region of Interest (ROI). From the TSPM, we derive three radiomic features, the TSPM entropy, uniformity, and mutual information (MI). The tissue signature first order statistics (TSFOS) features, which characterize the distribution of voxel intensities across all the imaging parameters are calculated, similar to traditional first order radiomics. The second order mpRad features are calculated from the tissue signature co-occurrence matrix features (TSCM). The TSCM characterizes the spatial relationship between tissue signatures within an image or ROI. 

##### Radiomic Parameters

We defined five mpRad and 51 single radiomic features to quantify the textural properties of the breast tumor on the post-contrast DCE MRI parameters [25,45,46,47,48,49]. The radiomic features can be sub divided into five categories: First Order Statistics (FOS, three–mpRad, 14 single features), Gray Level Co-occurrence Matrix (GLCM, two-mpRad, 18 single features), Gray Level Run Length Matrix (GLRLM, 11 single features), Neighborhood Gray Tone Difference Matrix (NGTDM, 5 single features), fractal dimension features (2 single features), and convexity. 

The input parameters were determined from empirical analysis for the statistical texture analysis methods of FOS, GLCM, GLRLM, and NGTDM were set as follows:

- Binning for FOS = 64.
- Gray level quantization for GLCM, GLRLM and NGTDM = 64.
- The distance d for GLCM was set to one voxel.
- Both GLCM and GLRLM were evaluated in all the four directions—0°, 45°, 90°, and 135°. Rotational invariance was achieved by extracting the radiomic features from GLCM and GLRLM averaged across all the directions.

We also computed radiomic feature maps (RFMs) [32] corresponding to the FOS and GLCM features. The mean of the RFM feature map metrics were used in the prediction model. Finally, the fractal dimension for the tumor intensity profile and tumor boundary were evaluated in addition to the convexity of the tumor boundary. All of the radiomic features were extracted using our in-house software developed using MATLAB (Version 19a).

#### 2.3.7. Patient Classification

We implemented patient classification using the hybrid IsoSVM feature transformation and classification algorithm [32] based on the Isomap [40] and the Support Vector Machine (SVM) algorithms [50]. We evaluated the following four models:

- Low vs. intermediate risk group.
- Low vs. high-risk group.
- Intermediate vs. high-risk group.
- Low vs. intermediate and high-risk groups combined.

The imbalance in the number of patients in different risk groups was overcome by setting different misclassification penalties for different risk groups while training the SVM [51]. The optimal values for the Isomap neighborhood parameter and the misclassification penalty were estimated using leave one out cross validation.

### 2.4. Multiparametric Breast Imaging

Patients were scanned on a 3T MRI system (3T Achieva, Philips Medical Systems, Best, The Netherlands) using a bilateral, dedicated four-channel, phased array breast coil (Invivo, Orlando, FL, USA) with the patient in the prone position. 

#### 2.4.1. Proton MRI Imaging

T_2_-weighted spin echo (Transverse Relaxation, Echo Time, Inversion Recovery (TR/TE/IR) = 7142/70/220 ms, Field of View (FOV) = 350 × 350, matrix = 220 × 195, slice thickness (ST) = 5 mm, Sensitivity encoding (SENSE) = 2, and Averages (Ave) = 2), and fast spoiled gradient echo (FSPGR).

T_1_-weighted (TR/TE = 5.4/2.3 ms, FOV = 350 × 350, matrix = 548 × 550, ST = 3 mm, SENSE = 2 and Ave = 1) sequences were acquired.

#### 2.4.2. Pharmacokinetic Dynamic Contrast-Enhanced MRI

The Pharmacokinetic (PK) DCE was obtained using non-fat-suppressed (FS), three-dimensional (3D), FSPGR T1-weighted (TR/TE = 3.4/1.7 ms, FOV = 350 × 350, matrix = 256 × 126, Flip angle (FA) = 10, slice thickness = 5 mm, and Ave = 1) sequences. Pre- and fourteen post- contrast images (temporal resolution = 15 s) after intravenous administration via a power injector at a rate of 2 mL/s of a gadolinium (Gd-DTPA) contrast agent (0.2 mL/kg (0.1 mmol/kg)).

#### 2.4.3. High-Resolution Dynamic Contrast-Enhanced MRI

T_1_-weighted 3D Gradient Recalled Echo (GRE) with FS (TR/TE = 5.8/2.9 ms, FOV = 350 × 350, matrix = 720 × 720, flip angle = 13, ST = 3 mm, and Ave = 1) were obtained pre and post the PK-DCE. 

#### 2.4.4. Diffusion Weighted Imaging

Diffusion-weighted imaging was acquired before contrast imaging using an FS fast spin echo planar parallel imaging sequence (TR/TE/IR = 9548/70 ms, FOV = 350 × 350, matrix = 220 × 195, SENSE = 2, Ave = 2, ST = 3 mm, b = 0, 200, 600, 800 s/mm^2^) on three planes and in less than three minutes. Trace apparent diffusion coefficient (ADC) maps were constructed using a diffusion monoexponential model. 

### 2.5. MRI Data Analysis 

#### 2.5.1. Clinical Breast Lesion Classification Methods 

Breast lesions were identified on the breast MRI by a radiologist and defined by the Breast Imaging-Reporting and Data System (BI-RADS) lexicon [52,53]. Breast density was defined as extremely dense tissue, heterogeneously dense tissue, scattered fibroglandular tissue, or primarily fatty tissue. Background parenchymal enhancement (BPE) was defined as minimal, mild, moderate, or marked. Lesions were classified as a focus, a mass or non-mass enhancement (NME). Morphologic assessment was defined for masses, as shape (round, oval, irregular), margins (1 = circumscribed, 2 = not circumscribed 2a = irregular 2b = spiculated), and enhancement patterns (1 = homogenous, 2 = heterogeneous or 3 = rim). For NME, distribution (1 = focal, 2 = regional, 3 = linear 4 = diffuse, and 5 = segmental) and enhancement pattern (homogenous, heterogeneous, clustered ring, and clumped) were recorded. We defined lesion morphology into seven classes (1 = focal NME, 2 = regional NME, 3 = linear or segmental NME, 4 = circumscribed mass, 5 = irregular mass, or 6 = spiculated mass). 

#### 2.5.2. Pharmacokinetic Contrast Enhancement Metrics

Pharmacokinetic kinetic DCE MRI provides quantitative metrics of the volume transfer constant (K^trans^ (min^−1^)), which characterize uptake of the contrast agent, the leakage within the extracellular extravascular space (Ve (%)), and the transfer rate constant (kep (min^−1^)). Post-processing of the DCE exam was performed by a combined Brix and Tofts model [15,54,55] using DynaCAD (Invivo, FL, USA) software from the identified breast lesions, and detailed in these manuscripts [11,56,57,58]. 

#### 2.5.3. ADC Mapping

Regions of Interest (ROI) were drawn on normal appearing glandular tissue and breast lesions defined by DCE MRI. Means and standard deviations were calculated for both tissue types. Ratios of lesion ADC to glandular tissue ADC (L/GT) were calculated from equation (6) below using the lesion and glandular tissue [23].

Normalized ADC value = (ADC value of Lesion)/(ADC value of glandular tissue).(6)

### 2.6. Statistical Analysis

We computed summary statistics (mean and standard deviation of the mean) for the quantitative imaging parameters from the mpMRI. An unpaired two-sided t-test was performed between each pair of risk groups imaging parameters to determine statistical significance. Sensitivity and specificity, and Area Under the Curve (AUC), were calculated to determine the classification of the patients in the different groups. Statistical significance was set at *p* ≤ 0.05.

## 3. Results

### 3.1. Clinical Demographics

A total of 80 patients with 83 lesions of 123 patients identified who had both multiparametric MRI imaging and Oncotype assay were selected. There were 19 (24%) lesions with Recurrence Score low risk (0–17), 49 (61%) lesions with intermediate risk (18–31), and 12 (15%) lesions with high risk (>31). Seventy-four patients were ER+/PR+ and six had only ER+ expression. The age distribution was low risk (age = 53 ± 10 years), intermediate risk (age = 53 ± 11 years) group, and high-risk (age = 54 ± 9 years) group. These data are summarized in Table 1. The 42 patients not included in the study did not undergo a complete advanced mpMRI imaging session and one patient that was HER2-neu positive. 

### 3.2. Radiological Findings

**Figure 2:**
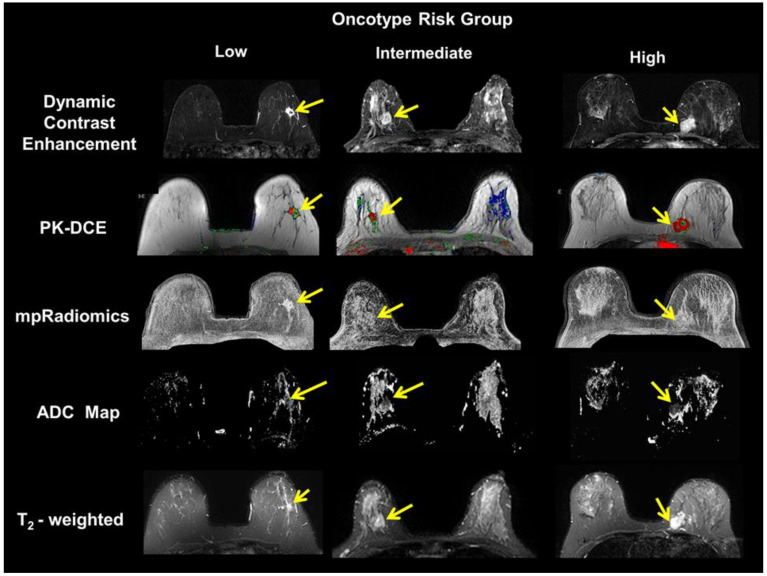
Demonstration of multiparametric magnetic resonance imaging (MRI) breast imaging of each risk group defined by the OncotypeDX. (**Left** Column) typical imaging of the low risk patient. (**Middle** Column) typical imaging of the medium risk patient. (**Right** Column) typical imaging of a high-risk patient. The yellow arrows indicate the primary lesion in the breast. The MRI images of T_2_-weighted (T_2_), apparent diffusion coefficient (ADC) map, Pharmacokinetic Dynamic Contrast Enhanced (PK-DCE) and multiparametric radiomics demonstrate the different malignant lesions.

**Figure 3:**
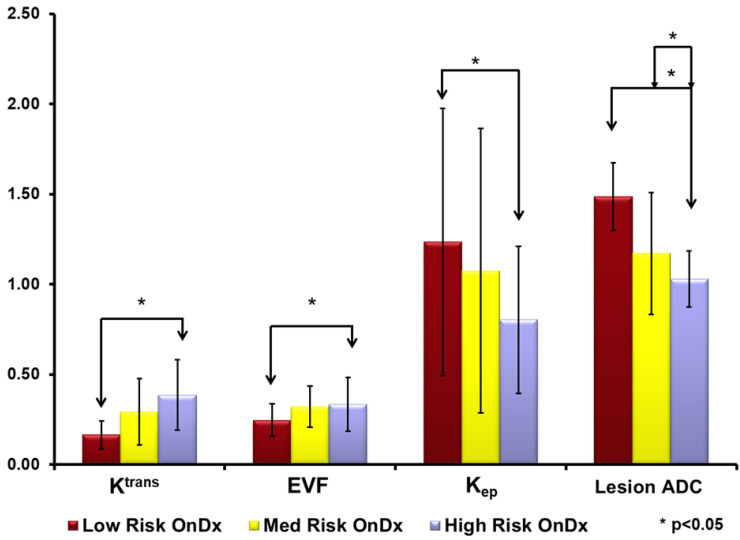
Bar graphs of quantitative multiparametric MRI parameters for each risk group from the OncotypeDX (OnDx). There are significant differences between each group of patients in the apparent diffusion coefficient (ADC (×10^−3^ mm^2^/s)) of water and the Pharmacokinetic Dynamic Contrast Enhanced (PK-DCE) metrics. The PK-DCE metrics are the volume transfer constant (*K*^trans^ (min^−1^)) and the fractional volume of the extracellular extravascular space (EVF (V*_e_*)). * *p* < 0.05.

Representative multiparametric breast imaging for each risk group as defined by OncotypeDX are illustrated in Figure 2. The high-risk group had the largest tumor size (2.9 ± 1.7 mm). Followed by the low-risk group tumor size (1.9 ± 1.3 mm) and the intermediate risk group (1.7 ± 1.4 mm). These differences were not significant (*p* > 0.5). For advanced MRI parameters, there were clear differences in each parameter and Oncotype risk groups. The PK-DCE parameter K^trans^ values for the intermediate- and high-risk groups were higher (0.46 and 0.49 (1/min) (*p* = 0.26)) compared to the low-risk group (0.30 (1/min) (*p* = 0.02). Similar results were noted for the other PK-DCE parameters (extracellular extravascular space (EVF) and k_ep_). The maximum contrast enhancement from DCE was largest for the low-risk group (503 ± 33 s), compared to the intermediate-risk (461 ± 24 s), and high-risk groups (468 ± 31 s). Similarly, the ADC map values from the high- and intermediate-risk patients in the lesion tissue were significantly lower (*p* < 0.05) than those for the low-risk patients (1.14 vs. 1.49 × 10^−3^ mm^2^/s). However, the ADC map values in glandular tissue remained constant across all groups (2.14–2.17 × 10^−3^ mm^2^/s). The bar graphs are shown in Figure 3.

### 3.3. Single and Multiparametric Radiomics

**Figure 4:**
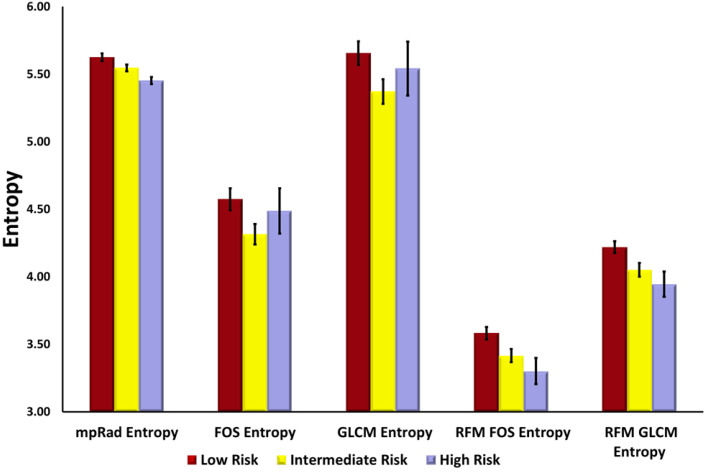
Bar graphs of different mpRad and single parameter entropy features extracted from DCE-MRI. Multiparametric radiomics (mpRad), Frist Order Statistics (FOS), Gray Level Co-occurrence Matrix (GLCM), radiomic feature mapping (RFM).

The single and multiparametric radiomic features demonstrated significant differences between the low, intermediate, and high-risk groups and shown in Figure 4. The single and multiparametric radiomic features, first order entropy feature map and the GLCM entropy feature map, which are measures of heterogeneity, were significantly higher for the low risk group as compared to the intermediate and high-risk groups. In contrast, the first order uniformity and GLCM energy, both of which are measures of homogeneity demonstrated an opposite trend. These radiomic results are summarized in Table 2. 

### 3.4. Integrated Radiomics Informatic System (IRIS) Model

**Figure 5:**
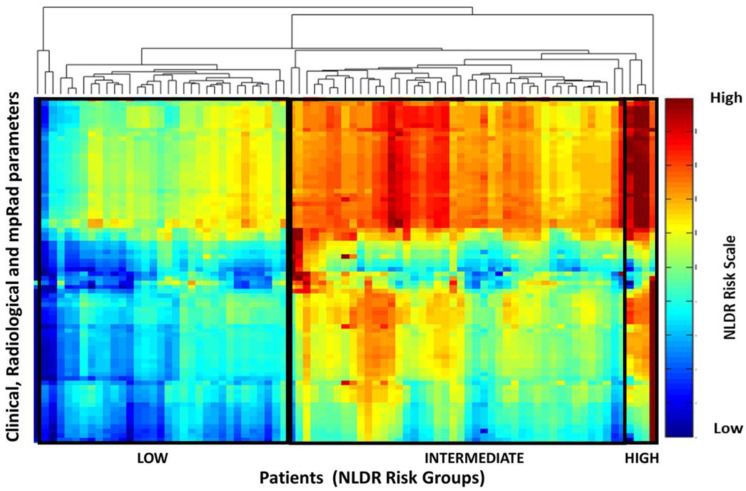
The IRIS heat map demonstrates the stratification of the integrated mpRad, radiological, and clinical parameters into OncotypeDX score recurrence risk groups of low, intermediate, and high. Interesting there appears to be intermediate and high-risk factors perceived in the low risk group that may not habeen noted in clinical evaluations. Similarly, there are a mix of low and high-risk features in the intermediate group. Integrated Radiomic Informatic System (IRIS), Non-linear dimension reduction (NLDR).

The IRIS heatmap risk profile of each patient is shown in Figure 5. The top surrogates for mpRad and single radiomics, imaging, and histological parameters determined from the clinical and imaging model based on the IRIS heatmap are summarized in Table 3 and Table 4.

**Figure 6:**
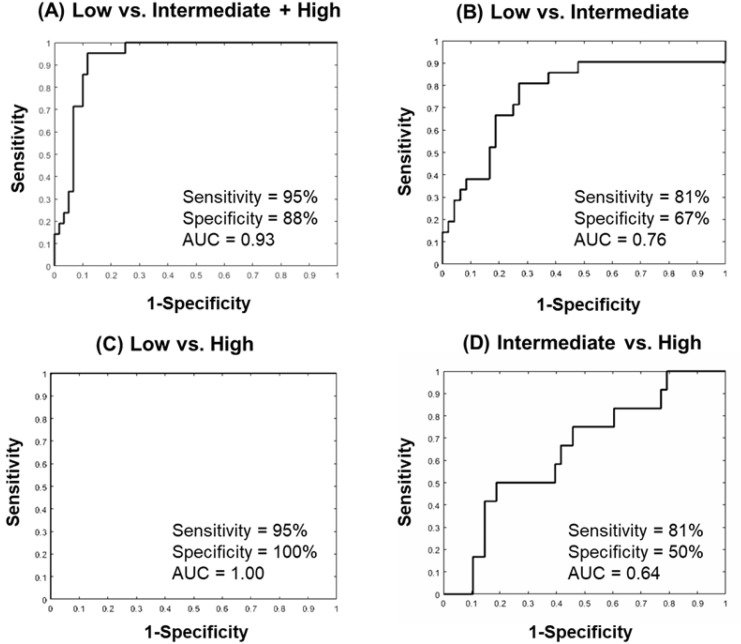
The Area under the Curve (AUC) graphs of the single IsoSVM model trained using quantitative imaging parameters (PK-DCE, ADC), Ki67, and single radiomics (FOS Entropy, Uniformity; GLCM Entropy, Energy) with the OncotypeDX score compared to each group. (**A**) The low versus intermediate + high-risk group. The sensitivity was 95% and specificity was 88% with an AUC = 0.93 (CI = 0.88–0.99). (**B**) The low versus intermediate risk group resulted in a sensitivity of 81%, specificity of 67%, and an AUC = 0.76 (CI = 0.63–0.90). (**C**) The low versus high risk group resulted in a sensitivity of 95%, specificity of 100%, and an AUC = 1.00 (CI = 1.00–1.00). (**D**) The intermediate versus high risk group resulted in markedly lower (non-diagnostic) AUC and other metrics with a sensitivity of 81%, and specificity of 50%, and an AUC = 0.64 (CI = 0.47–0.81). Gray Level Co-occurrence Matrix (GLCM).

**Figure 7:**
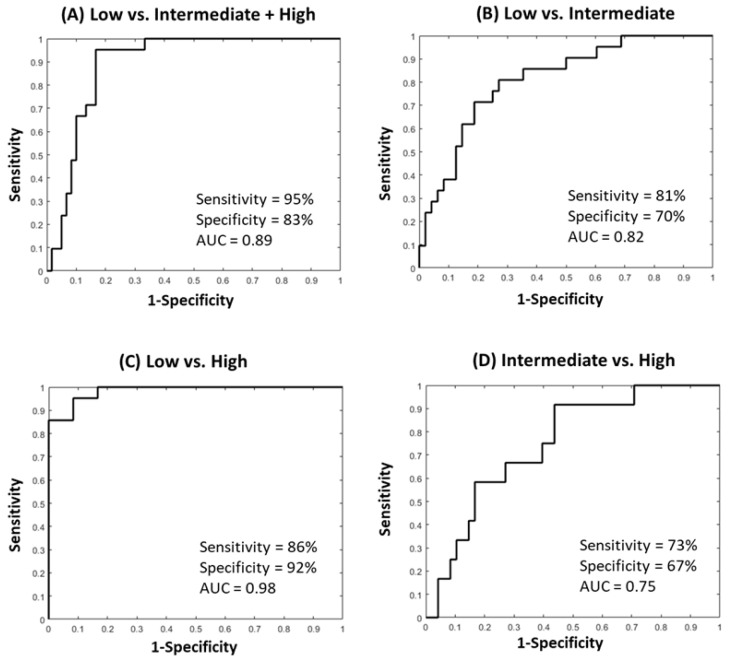
The Area under the Curve (AUC) graphs of the mpRad IsoSVM model trained using quantitative imaging parameters (PK-DCE, ADC), Ki67, and mpRad (TSFOS Entropy, Uniformity, Energy) with the OncotypeDX score compared between each group. (**A**) The low versus intermediate + high-risk group. The sensitivity was 95% and specificity was 83% with an AUC = 0.89 (CI = 0.82–0.96). (**B**) The low versus intermediate risk group resulted in a sensitivity of 81%, specificity of 70%, and an AUC = 0.82 (CI = 0.7–0.92). (**C**) The low versus high risk group resulted in a sensitivity of 86%, specificity of 92%, and an AUC = 0.98 (CI = 0.95–1.00) and class imbalance of 3:1. (**D**) The intermediate versus high risk group resulted in markedly lower (non-diagnostic) AUC and other metrics with a sensitivity of 73% and specificity of 67% and an AUC = 0.75 (CI = 0.61–0.89). Tissue signature First Order Statistics (TSFOS).

For the patient classification, the quantitative imaging parameters, ADC map values and the PK-DCE parameters were combined with histopathological parameter of Ki-67, and the radiomic parameters of FOS/GLCM entropy, FOS uniformity, and GLCM energy features. For the single parameter-based IRIS model, the IsoSVM model classified the low risk group from the intermediate and high-risk groups with a sensitivity and specificity of 95% and 88%, respectively, with an AUC of 0.93 (CI = 0.88–0.99). The optimal neighborhood parameter, dimensionality, and imbalance ratio were found at 60, 10, and 2:1, respectively. The ROC curves from different inter-group IsoSVM classifiers are shown in Figure 6. The lowest and non-diagnostic AUC (0.64 (CI = 0.47–0.81)) resulted from the comparison between the intermediate and high-risk groups. Figure 7 demonstrates the ROC curves from different inter-group IsoSVM classifiers using mpRad based IRIS model. There was no significant difference between the ROC curves from the two radiomic feature sets. The p values for comparison between the ROC curves for single and multiparametric radiomics were *p* = 0.33 (low vs. intermediate + high), *p* = 0.49 (low vs. intermediate), *p* = 0.30 (low vs. high), and *p* = 0.19 (intermediate vs. high).

**Figure 8:**
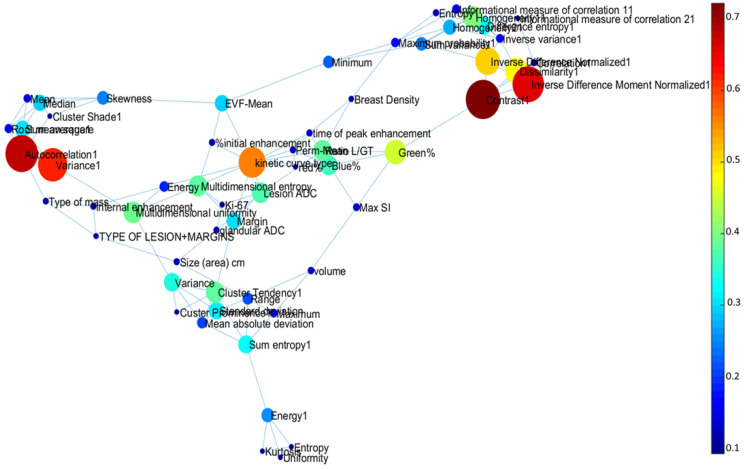
Visualization of the Integrated Radiomic Informatic System (IRIS) single parameter radiomics complex interaction network. The nodes represent different IRIS parameters, with edges corresponding to inter-parametric relationships.

**Figure 9:**
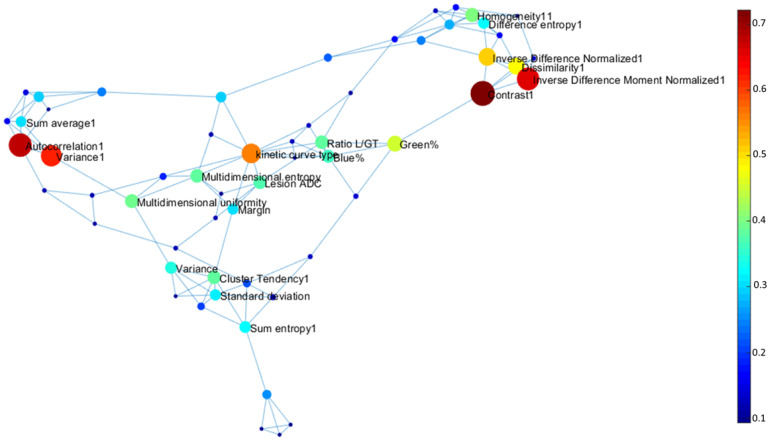
Visualization of the Integrated Radiomic Informatic System (IRIS) mpRad complex interaction network. The nodes represent different IRIS parameters, with edges corresponding to inter-parametric relationships.

The topological graph theoretic metrics for integrated centrality and other different centrality measures for each informatics parameter are summarized in Table 4 and Table 5. The average path length between each parameter was 2.1 and diameter of the complex informatics network was 5.1. The average clustering coefficient was 0.53, much higher than the clustering coefficient of Erdos–Renyi random graph (CCER = 0.0228). Figure 8 and Figure 9 illustrate the complex interaction network and the inter-parametric relationships between all the variables for single and multiparametric radiomics models. The hub IRIS parameters for single and multiparametric radiomics IRIS models have been summarized in Table 5 and Table 6.

## 4. Discussion

We have introduced and demonstrated an advanced NLDR integrated clinical and imaging model (IRIS) to analyze the relationships and interactions between mpMRI parameters, radiomics, clinical heath records, and histological variables and compared these results with the OncotypeDX assay for risk assessment of breast cancer recurrence. The data integration by the IRIS model using radiomic, radiological, and clinical variables were able to group patients into the three different categories of low, intermediate, and high risk of breast cancer recurrence. Importantly, using IRIS, we defined several mpMRI and mpRad variables that were predictive of potential local and systemic tumor recurrence compared with categorization as defined by the OncotypeDX risk score. This integration of mpMRI, clinical data, and radiomics compared favorably to OncotypeDX and may lead to an accurate non-invasive assessment for risk of local and systemic recurrence. For example, we found that the most important radiological and histological parameters were the ADC map values, PK-DCE metrics, and Ki-67. These quantitative biological metrics reflect the cellularity, vascularity, and proliferation status of the tumor. Therefore. By using an integrated machine learning model of imaging, radiomics, clinical heath records, and histopathology data has the potential to describe unseen features of cancer and may provide data for precision personalized care as shown from the IRIS visualization heatmap. This is the one of the first studies to use an integrated graph theoretic and machine learning model based on quantitative mpMRI, clinical variables, and both single and multiparametric radiomics in breast cancer compared with gene array data [35,37].

The categorization of the different risk groups from our model was strikingly consistent based on the combined imaging, radiomics, and pathological variables. Indeed, the ADC map values were lower in the high and intermediate risk group consistent with the increased cell proliferation metric, Ki-67 from histological analysis. The PK-DCE parameters and lesion size demonstrated similar characteristics in these groups. Moreover, the radiomics parameters revealed an interesting underlying pattern across each of the three risk groups.

This report demonstrates that using an advanced unsupervised machine learning method in breast cancer and integration of several variables can accurately separate those cancers into different risk stratifications consistent with the OncotypeDX results. Finally, by using the complex interaction mapping, one can visualize the connections formed between each variable, which may form the basis for even further predictive modeling using the heatmap visualization tool or newer surveillance methods of recurrence in clinical use. For example, in some low risk patients, there were potential markers of increased risk, which may be missed by just looking at “single points” of data and these cases could be followed more closely over time. Similarly, potentially high-risk markers were seen in the intermediate risk cases, which could be useful for a surveillance program in these patients.

The clinical and radiological parameters utilized in this study were derived from our clinical experience, since current treatment decision algorithms are based on standard clinicopathologic prognostic, and predictive factors, large datasets using clinical measures such as tumor size, node status, grade, ER, and HER2-neu [9,10,59,60,61,62,63]. Similarly, imaging features such as, breast density, lesion morphology, size, enhancement patterns as well as quantitative metrics (ADC map values and PK-DCE) are routinely used in practice and are familiar to the radiologist and readily available. Finally, radiomic feature analysis methods are increasing bringing a new potential quantitative biological biomarker to different cancers. The OncotypeDX assay has been shown to be a predictive tool to identify patients most likely to benefit from the addition of adjuvant chemotherapy to endocrine therapy with validation in prospective studies [1,2,3]. Thus, the ability to combine these quantitative measures would be an important step in ensuring that “the right patient receives the right treatment”.

We developed an integrated informatics-radiomics decision support system (IRIS) based on multi-subspace embedding and clustering method for the purpose of diagnosis or prognosis [36]. Furthermore, the complete multi-subspace embedding, and clustering method is unsupervised and does not require any training data. The IRIS heatmap provides a visualization of relationship between different cancers along with quantifiable embedding metrics. Using the IRIS heatmap, we would be able to identify a patient or a group of patients with the most similar informatics embedding metrics and use these metrics for a new patient with an unknown risk of recurrence. Understanding the complex relationships between different embeddings can provide an insight on how these metrics are related at biological level predicting recurrence of breast cancer. Interestingly, the lesion size was not an accurate feature for categorizing cancers into the risk groups in this study. The lesion size was largest for the highest risk group, but was smallest for the intermediate risk group, suggesting that lesion size alone is not an accurate predictor. Differentiating and characterizing benign from invasive breast cancer is an important issue that was the focus of many different studies [12,19,63,64,65,66].

The complex interaction network provided insight into how the different IRIS parameters relate to each other. For example, radiomic features of entropy and uniformity have higher integrated centrality in the subgraph indicating these features provided complementary information about the underlying network. In contrast, glandular ADC and Ki-67 have lower integrated centrality and more associated with other distinct features in the network. The highest integrated centrality measures were the mpRad and vascular features suggested the mpMRI provides more information about breast lesions.

Prior studies developing risk scores comparable to the OncotypeDX, typically used a single or just few MRI parameters [10,34,67,68]. Some differences with those studies and the present study are several; we employed machine learning and graph theory methods to differentiate these entities. The IRIS model incorporated pathophysiological and imaging characteristics of different breast tissue types, enabling a more predictive model of the tumor environment. Finally, we used the standard of care BI-RADS information from the radiologist report in clinical records, which is an informatic risk assessment and provides diagnostic characteristics of the lesion and surrounding tissue. The addition of BI-RADS increased the dimensionality to our model by the combination of the breast density, mass shape, margins, enhancement patterns, and other factors, in conjunction with the radiomics features, a more complete picture of the lesion and surrounding texture characteristics. One limitation to the study is our small sample size of patients, but this report provides encouraging preliminary data for further testing on a larger cohort in a prospective study. For example, the non-diagnostic AUC between the intermediate and high-risk group in this study may be attributed to the large class imbalance of one to seven between the groups. Recent reports have shown non-diagnostic AUCs (0.50–0.77) results [69] in a larger group of similar patients studied using the OncotypeDX and single parameter radiomics consistent with our results. However, the authors in those studies used only a single MRI parameter (DCE) and developed some different features based on image analysis, but they did not utilize quantitative PK-DCE, ADC mapping, or other common MRI parameters, which are more representative of the tumor microenvironment. 

## 5. Conclusions

The incorporation of multiparametric radiomics into the IRIS model provided more quantitative metrics for better characterization and complete picture of breast lesions. Ongoing work is underway to see if IRIS could be used as a potential predictive or selection tool for patients for adjuvant treatment and assist in decision making with pathology for those tough cases in the “low to intermediate” risk group. IRIS could be used to identify high-risk features in the low to intermediate groups for potential active surveillance, in terms of risk defined by the integration of several variable over time.

In conclusion, these initial studies provide insight into the molecular underpinning of the surrogate imaging and clinical features and provide the foundation to relate these changes to histologic and molecular pathology parameters. The integration of these clinical and imaging parameters may help refine available prognostic and predictive markers, and improve clinical decision-making.

## 6. Patents

Jacobs, M.A.; Parekh, V.S. (IRIS): Integrated Radiomic Informatic System: A novel informatics radiomics method for the integration of many types of data for classification into different groups. 20170112459. 27 April 2017.

## Figures and Tables

**Table 1:** Patient clinical parameters and demographics.

Clinical Information	Mean ± SD
Patient Age	53 ± 10
Tumor Grade (Elson)	
1	8
2	60
3	12
Ki67 (%)	29 ± 16
ER+	80
PR+	74
* HER2-neu+	0
Triple Negative	0
ODX Score	
Low (≤17%)	19
Intermediate (18–30%)	49
High (≥31%)	12

Estrogen Receptor (ER), Progesterone Receptor (PR), HER2-neu + (human growth factor receptor 2) * HER2-neu patients were excluded. ODX = OncotypeDX; Ki-67 is a histological marker for proliferation. Standard Deviation = SD; HER2 = human growth factor receptor 2; * HER2-neu patients were excluded.

**Table 2:** Summary of entropy and uniformity radiomic features characterizing the heterogeneity or homogeneity of breast tumors across the different risk categories.

Radiomic Features	Low Risk	Intermediate Risk	High Risk	*p*-Value *
mpRad FOS Energy	1359 ± 223	1346.45 ± 171	3332 ± 900	0.03
mpRad FOS Entropy	5.62 ± 0.03	5.55 ± 0.02	5.45 ± 0.03	0.009
mpRad FOS Uniformity	0.02 ± 0.001	0.03 ± 0.001	0.03 ± 0.001	0.004
FOS Entropy	4.57 ± 0.09	4.32 ± 0.08	4.49 ± 0.17	0.06
FOS Uniformity	0.05 ± 0.003	0.06 ± 0.003	0.06 ± 0.007	0.01
GLCM Entropy	5.67 ± 0.10	5.37 ± 0.09	5.54 ± 0.20	0.04
GLCM Energy	0.01 ± 0.000	0.01 ± 0.001	0.01 ± 0.002	0.002
RFM FOS Entropy	3.58 ± 0.05	3.42 ± 0.05	3.30 ± 0.10	0.006
RFM FOS Uniformity	0.10 ± 0.004	0.12 ± 0.005	0.13 ± 0.009	0.004
RFM GLCM Entropy	4.23 ± 0.05	4.05 ± 0.05	3.95 ± 0.09	0.004
RFM GLCM Energy	0.02 ± 0.001	0.03 ± 0.003	0.03 ± 0.003	0.003

* low vs. intermediate high-risk groups, Multiparametric radiomics (mpRad), Frist Order Statistics (FOS), Gray Level Co-occurrence Matrix (GLCM), radiomic feature mapping (RFM).

**Table 3:** The ranking of single radiomic features by IRIS.

Parameter	Rank
Ki-67	1
Ratio Lesion/Glandular Tissue	2
Lesion ADC	3
%initial enhancement	4
RFM FOS kurtosis	5
Perm-Mean	6
NGTDM Busyness	7
GLCM Correlation	8
GLRLM GLN	9
GLRLM RLN	10
RFM Laplacian	10
RFM LoG	10

Frist Order Statistics (FOS), Gray Level Co-occurrence Matrix (GLCM), Gray Level Run Length Matrix (GLRLM), Gray Level Nonuniformity (GLN), Run Level Nonuniformity (RLN), radiomic feature mapping (RFM), Neighborhood Gray Tone Difference Matrix (NGTDM).

**Table 4:** The ranking of mpRad features identified by IRIS.

Parameter	Rank
Ratio L/GT	1
Lesion ADC	2
%initial enhancement	3
TSFOS Energy	4
TSFOS Kurtosis	5
TSPM Multidimensional uniformity	6
TSFOS Minimum	7
TSCM Dissimilarity1	8
TSCM Inverse Difference Normalized1	9
TSCM Inverse variance1	10
TSCM Sum variance1	10

Integrated Radiomic Informatic System (IRIS), Multiparametric radiomics (mpRad), Frist Order Statistics (FOS), Gray Level Co-occurrence Matrix (GLCM), Gray Level Run Length Matrix (GLRLM), Gray Level Nonuniformity (GLN), Run Level Nonuniformity (RLN), radiomic feature mapping (RFM), Neighborhood Gray Tone Difference Matrix (NGTDM), Tissue signature Frist Order Statistics (TSFOS), Tissue signature probability matrix (TSPM), Tissue signature co-occurrence matrix features (TSCM).

**Table 5:** Summary of centrality values across different IRIS parameters.

IRIS Parameter	Betweenness Centrality	Degree Centrality	Harmonic Centrality	Integrated Centrality
GLCM Variance	246	5	347.84	0.58
GLCM Autocorrelation	167	4	357.07	0.53
RFM FOS uniformity	886	7	3.55	0.53
NGTDM Contrast	1115	5	0.82	0.52
GLCM Energy	1084	5	1.35	0.51
RFM GLCM entropy	763	7	4.86	0.49
GLCM Maximum probability	975	5	1.32	0.48
GLCM Homogeneity 2	860	5	10.34	0.45
GLCM Correlation	747	6	0.83	0.45
kinetic curve type	371	9	0.66	0.44

Integrated Radiomic Informatic System (IRIS), Frist Order Statistics (FOS), Gray Level Co-occurrence Matrix (GLCM), Gray Level Run Length Matrix (GLRLM), Gray Level Nonuniformity (GLN), Run Level Nonuniformity (RLN), radiomic feature mapping (RFM), Neighborhood Gray Tone Difference Matrix (NGTDM).

**Table 6:** Summary of centrality values across different IRIS parameters with mpRad.

IRIS parameter	Betweenness Centrality	Degree Centrality	Harmonic Centrality	Integrated Centrality
TSCM Contrast I	406.00	5.00	150.18	0.72
TSCM Autocorrelation I	238.00	5.00	210.53	0.68
TSCM Inverse Difference Moment Normalized1	367.00	4.00	150.18	0.66
TSCM Variance I	195.00	4.00	210.52	0.61
kinetic curve type	280.00	11.00	0.84	0.56
TSCM Inverse Difference Normalized1	358.00	6.00	21.24	0.51
TSCM Dissimilarity I	357.00	5.00	21.25	0.48
Green %	399.00	4.00	0.77	0.45
TSCM Homogeneity II	202.00	7.00	15.00	0.40
TSPM uniformity	330.00	4.00	0.99	0.39

Integrated Radiomic Informatic System (IRIS), Multiparametric radiomics (mpRad), Frist Order Statistics (FOS), Gray Level Co-occurrence Matrix (GLCM), Gray Level Run Length Matrix (GLRLM), Gray Level Nonuniformity (GLN), Run Level Nonuniformity (RLN), radiomic feature mapping (RFM), Neighborhood Gray Tone Difference Matrix (NGTDM), Tissue signature Frist Order Statistics (TSFOS), Tissue signature probability matrix (TSPM), Tissue signature co-occurrence matrix features (TSCM).

## Appendix A

### Appendix A.1. Theoretical Graph Metrics

#### Appendix A.1.1. Clustering Coefficient

The clustering coefficient defines the connectedness of the neighborhood of an informatics parameter. Clustering coefficient ranges from zero to one with zero representing completely disconnected neighborhood and one representing completely connected neighborhood. Mathematically, the clustering coefficient, *i* is defined by Equation (A1),

(A1) $$CC\left(i\right)=\;\frac{2e_{i}}{k_{i}\left(k_{i}-1\right)},$$

Here *e_i_* is the number of connected edges neighborhood of *i*, *k_i_* are the number of nodes in the neighborhood of *i* making $\frac{k_{i}\left(k_{i}-1\right)}{2}$ the maximum possible number of edges in the neighborhood.

#### Appendix A.1.2. Degree Distribution

The degree distribution is the most fundamental metric calculated for any complex network. The degree distribution, *P*(*k*) is determined using the following equation

(A2) $$P\left(k\right)=\frac{1}{N}\sum_{i=1}^{N}1\left\{\deg\left(i\right)=k\right\}\;\forall k\in \left\{7,8,\ldots ,N-1\right\},$$

Here, *deg*(*i*) represents the degree of the parameter, *i*, which is defined as the total number of parameters that are directly connected to it; *N* is the number of parameters.

The degree distribution enables us to identify whether the network is a scale-free network or not. For scale free networks, the degree distribution follows the power law $P\left(k\right)\propto k^{-\gamma }$, with the value of γ ranging between 2 and 3. Scale free networks are characterized by the presence of few highly connected hub nodes that influence the network properties and may correspond to the key parameters that are predictors of breast cancer recurrence risk [42].

### Appendix A.2. Centrality Measures

The Centrality measures determine the importance of each informatic parameter in the complex network. The most widely used measures of centrality are betweenness, harmonic, and degree centrality [43,44,70]. We use three centrality measures, each centrality measure highlighting a different importance property of the network.

#### Appendix A.2.1. Betweenness Centrality

Betweenness centrality quantifies the amount of information that flows through each parameter or node [43]. It is defined as the number of shortest paths that pass through a parameter, given by Equation (A3):

(A3) $$C_{B}\left(i\right)=\;\sum_{s\neq i\neq t}\frac{N_{st}\left(i\right)}{N_{st}},$$

Here, *N_st_* is the total number of shortest paths between the parameters, *s* and *t* and *N_st_*(*i*) is the total number of shortest paths between *s* and *t* that pass through *i*.

#### Appendix A.2.2. Harmonic Centrality

Harmonic centrality of a parameter is defined as the sum of inverse of all geodesic distances (path lengths) from that parameter to all the other parameters [44]. Harmonic centrality measures the nodes that are influential and how information spreads on the local network. Mathematically, harmonic centrality of a parameter, *i* is given by Equation (A4):

(A4) $$C_{H}\left(i\right)=\frac{1}{N-1}\sum_{\begin{array}{c} s=1\\ s\neq i \end{array}}^{N}\frac{1}{G\left(s,i\right)},$$

Here *G*(*s*,*i*) is the geodesic distance or path length between the parameters *s* and *i*.

#### Appendix A.2.3. Degree Centrality

Degree centrality or degree is defined as the total number of parameters linked to a node, i.e., the number of neighbors, where each parameter is connected in the complex network.

#### Appendix A.2.4. Integrated Centrality

Each centrality measure signifies the importance of each parameter based on a pre-define characteristic. Where, the significance of each parameter across all centrality indices can be defined using integrated centrality [17] as shown in Equation (A5):

(A5) $$C_{int}\left(i\right)=\frac{1}{3}\left(\frac{C_{B}\left(i\right)}{max\left(C_{B}\right)}+\;\frac{C_{H}\left(i\right)}{max\left(C_{H}\right)}+\;\frac{C_{D}\left(i\right)}{max\left(C_{D}\right)}\right),$$

Here, *C_int_* corresponds to the integrated centrality of the parameter, *i*; *C_B_*, *C_H_*, and *C_D_* represent the betweenness, harmonic, and degree centralities of the parameter, *i* respectively and *max* () extracts the maximum values of each centrality measure across all the parameters.
